# Diagnostic value of cardiac magnetic resonance for the differential diagnosis of thrombus vs tumor

**DOI:** 10.1186/1532-429X-15-S1-P103

**Published:** 2013-01-30

**Authors:** Pablo Pazos, Eduardo Pozo, Ines Garcia-Lunar, Adam Jacobi, Frank Macaluso, Valentin Fuster, Jagat Narula, Javier Sanz, Matthew D Cham

**Affiliations:** 1 The Mount Sinai Cardiac MRI/CT Program, Mount Sinai School of Medicine, New York, NY, USA

## Background

Correct differentiation of a cardiac tumor from a thrombus remains important in clinical practice as the prognosis and therapeutic approach vary substantially. Techniques such as echocardiography may have limitations due to reduced image quality and poor tissue characterization. Cardiac magnetic resonance (CMR) has emerged as a promising tool in this regard; however, studies assessing its value are scarce.

## Methods

We retrospectively analyzed the CMR of patients with a definite cardiac mass. Thrombus was defined as a non-infiltrating mass that fulfilled any of the following criteria: a) adjacent to an akinetic or dyskinetic myocardial segment (typically infarcted) or central catheter, b) located in the atrial appendage in patients with atrial fibrillation, c) experienced a significant reduction in size under anticoagulation therapy, or d) had surgical and/or pathological confirmation. Tumor was defined as a mass that did not fulfill any of the above criteria plus a) was infiltrative, or b) located in the left aspect of the fossa ovalis with typical features of myxoma, or c) had surgical and/or pathological confirmation. Mass features on common CMR sequences (cine image, T1-weighted [T1w] and T2 weighed [T2w] spin-echo, contrast first pass perfusion, post-contrast TI scout, and late gadolinium enhancement (LGE)) were analyzed. Categorical data were summarized as frequencies and percentages, and continuous variables were expressed as mean ± SD. Difference in CMR characteristics between thrombi and tumors were compared by Chi-square, Fisher's or t-student tests as appropriate. A two sided P-value of <0.05 was considered significant.

## Results

Fifty patients (26 with thrombus and 24 with tumors) were included. 30 (60%) were male and the mean age was 60 ±17 years. Gadolinium contrast agents were used in 48 (96%) patients. There was no difference in mass motility between thrombi and tumors. Thrombi were smaller, and more homogeneous than tumors. There were no significant differences in signal intensity on T1w images in comparison with the myocardium, whereas tumors were more frequently hyperintense on T2w images. Both perfusion and LGE were more common in tumors than in thrombi. A pattern of hyper-isointensity (in comparison with normal myocardium) with short inversion times and hypointensity with long inversion times on the TI scout had the highest diagnostic accuracy (93%) for the differentiation of tumor vs. thrombus.

## Conclusions

CMR is a useful technique for the differential diagnosis between cardiac thrombus and tumor and might be helpful in difficult cases. A typical pattern of mass hyper-isointensity with short inversion times and hypointensity with long inversion times has the highest diagnostic accuracy.

## Funding

No funding

**Table 1:** CMRI features of thrombus vs tumor

	Thrombus	Tumor	p	Sensitivity	Specificity	Accuracy
Size (mm) *	23±13	42±27	0.01	76%	71%	74%
Homogeneity (+)	23 (89%)	9 (39% )	<0.0001	89%	61%	76%
Motility (+)	5 (19% )	9 (38%)	0.15			
Hyperintensity T1w	2 (17%)	7 (29%)	0.69			
Hyperintensity T2w	8 (42%)	17 (74%)	0.04	58%	74%	67%
Perfusion (+)	2 (13% )	15 (68%)	0.001	87%	68%	76%
LGE (+)	1 (4%)	16 (67%)	<0.0001	96%	67%	81%
Typical pattern on TI scout**	19 (95% )	2 (9%)	<0.0001	95%	91%	93%

*Maximal diameter. Cutoff value ≥29.5 mm ** Hyper-isointensity (in comparison with normal myocardium) with short inversion times and hypointensity with long inversion times.

**Figure 1:**
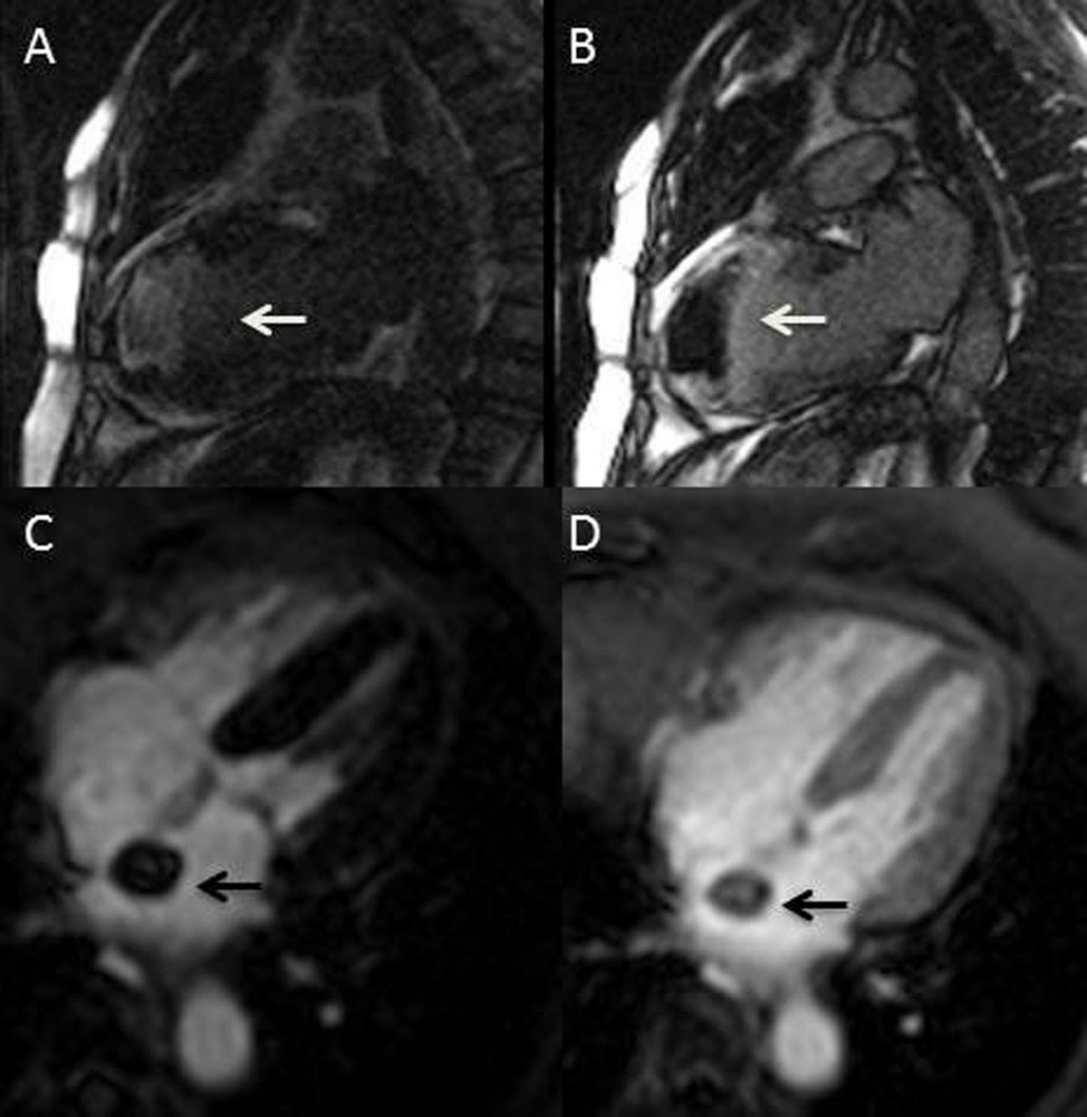
TI scout sequence in a case of thrombus (A and B, white arrow) and in a case of tumor (C and D, black arrow). In A and C the selected TI is short and in B and D is long. Note the typical pattern (hyperintensity-hypointensity) of the thrombus opposite to the tumor.

